# Supplementary material for: A Prospective Real-World Multi-Center Study to Evaluate Progression-Free and Overall Survival of Radiotherapy with Cetuximab and Platinum-Based Chemotherapy with Cetuximab in Locally Recurrent Head and Neck Cancer
Source: Cancers (Basel). 2021 Jul 8;13(14):3413. doi: 10.3390/cancers13143413 (PMC8305667; doi:10.3390/cancers13143413)
Supplement: Supplementary file 1 [file cancers-13-03413-s001.zip › cancers-1239148-supplementary.pdf]

cancers

MDPI

Supplementary Materials: A Prospective Real-World Multi-Center Study to Evaluate Progression-Free and Overall Survival of Radiotherapy with Cetuximab and Platinum-Based Chemotherapy with Cetuximab in Locally Recurrent Head and Neck Cancer

Markus Hecht, Dennis Hahn, Philipp Wolber, Matthias G. Hautmann, Dietmar Reichert, Steffi Weniger, Claus Belka, Tobias Bergmann, Thomas Göhler, Manfred Welslau, Christina Große-Thie, Orlando Guntinas-Lichius, Jens von der Grün, Panagiotis Balermpas, Katrin Orlowski, Diethelm Messinger, Karsten G. Stenzel, and Rainer Fietkau

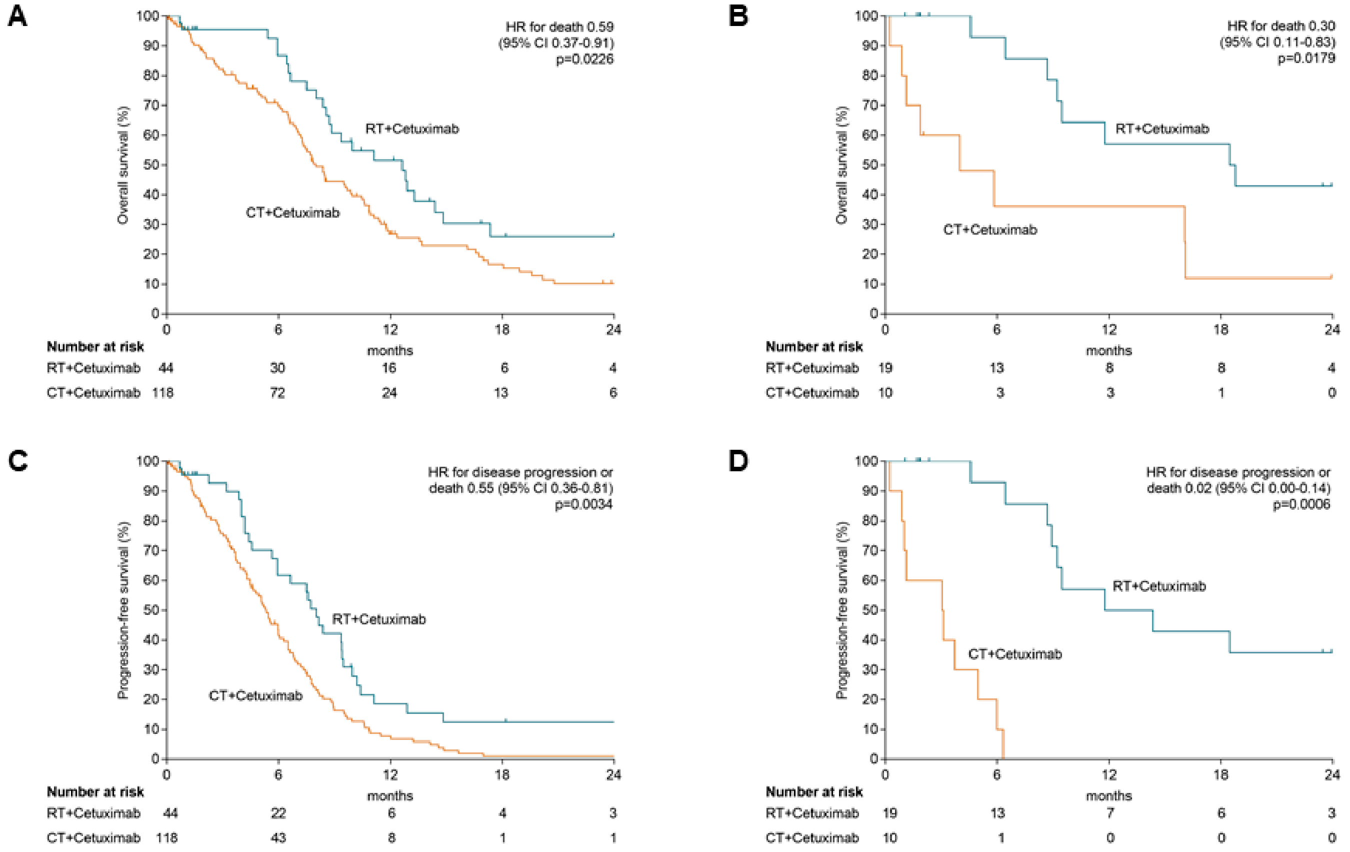

Supplementary Figure S1. Kaplan-Meier analyses of OS in patients with prior RT (**A**) and without prior RT (**B**) and PFS in patients with prior RT (**C**) and without prior RT (**D**).

Supplementary Table S1. Univariate and multivariable Cox proportional hazard models to investigate the association between patient characteristics and overall survival.

Explanatory factors		N	Death	HR	95% CI	*p*-value
		**Univariate**				
Age at study inclusion	≤ 65 years	113	76	1		-
	> 65–75 years	52	33	0.82	0.53–1.22	0.3333
	> 75 years	27	21	1.07	0.65–1.71	0.7715
Sex	Male	153	107	1		-

	Female	39	23	0.80	0.50–1.24	0.3423
Body weight *	Per 5 kg	192	130	1.06	0.99–1.13	0.1043
Alcohol consumption *	Never	44	34	1		-
	Several times per month	44	30	0.70	0.42–1.15	0.1575
	Several times per week or daily	47	31	0.59	0.36–0.95	0.0316
	Missing	57	35	0.66	0.41–1.06	0.0819
Smoking habits *	Never smoked	55	39	1		-
	Former smoker	72	50	0.98	0.64–1.50	0.9190
	Current smoker	64	40	0.66	0.43–1.03	0.0688
Charlson comorbidity index at study inclusion	0	95	62	1		-
	1	37	27	1.12	0.70–1.74	0.6344
	> 1	60	41	1.20	0.80–1.78	0.3611
Chemotherapy/Radiotherapy *	Chemotherapy only	129	98	1		-
	Radiotherapy only	63	32	0.50	0.33–0.75	0.0008
Duration since initial diagnosis	Per year	192	130	1.00	0.97–1.03	0.8195
Prior Radiotherapy *	Yes	163	114	1		-
	No	29	13	0.64	0.37–1.06	0.1013
Prior chemotherapy concomitant to radiotherapy *	Yes	109	77	1		-
	No	82	52	0.88	0.62–1.25	0.4925
ECOG performance status at treatment initiation *	0	31	17	1		-
	1	112	77	1.59	0.97–2.79	0.0828
	≥ 2	36	28	2.36	1.30–4.41	0.0054
	Unknown	13	8	1.16	0.45–2.68	0.7463
Location of primary tumor	Oropharynx	44	32	1		-
	Hypopharynx	33	24	1.06	0.61–1.81	0.8326
	Larynx	25	16	1.09	0.58–1.95	0.7902
	Oral cavity	61	38	0.98	0.61–1.58	0.9419
	Other	9	7	1.03	0.42–2.21	0.9344
	Multiple locations	20	13	1.38	0.70–2.57	0.3330
Overall VAS score *	Per 10 points	137	87	1.19	1.08–1.31	0.0004
**Multivariable †**						
Body weight	Per 5 kg	136	86	1.12	1.03–1.21	0.0054
Chemotherapy/Radiotherapy	Chemotherapy only	85	61	1		-
	Radiotherapy only	51	25	0.57	0.35–0.92	0.0240
Overall VAS score	Per 10 points	136	86	1.19	1.08–1.32	0.0008

HR: Hazard Ratio; CI: Confidence Interval; ECOG: Eastern Cooperative Oncology Group; VAS: Visual Analogue Scale. † Final Cox regression model after backward selection. Within the selection process, all explanatory factors with an effect *p*-value of < 0.2 in the univariate Cox regression analysis were considered (*). Only factors with $p < 0.05$ remained in the final model. Note: Due to missing data, mainly for the Overall VAS score, only 136 patients were available for the multivariable analysis.

Supplementary Table S2. Best Overall response during treatment.

	**RT + Cet** *n* = 63 **Patients (%)**	**CT + Cet** *n* = 129 **Patients (%)**	***p*-value**
Overall response (CR + PR)	26 (41)	33 (26)	0.0282
Complete response (CR)	9 (14)	8 (6)	

Partial response (PR)	17 (27)	25 (19)
Stable disease (SD)	8 (13)	37 (29)
Progressive disease (PD)	7 (11)	22 (17)
unknown	22 (35)	37 (29)
